# Medical News and Miscellany

**Published:** 1900-01

**Authors:** 


### Medical News and Miscellany.

---

**Dr. S. G. Harper,** late of Hutto, has located in Austin.

---

**Dr. F. A. Young** hos removed from Sublime, to Montgomery, Texas.

---

**Dr. E. C. Partee** has removed from Laughlin, Ark., to Magnolia, Ark.

---

**Dr. J. Z. Ferrell** has removed from Mt. Sylvan, Texas, to Tyler, Texas.

---

**Dr. R. C. Youngblood** has removed from McCoy, Texas, to Waco, Texas.

---

**Dr. Andrew Sibley** has removed from Palestine, Texas, to Dublin, Texas.

---

**Dr. A. J. Gilbert** has removed from Prairie Hill, Texas, to Hillsboro, Texas.

**Dr. Ed. D. Batts,** recently House Surgeon Sealy Hospital, Galveston, has removed to Houston.

---

**Dr. Robert Dinwiddie,** of San Antonio, Texas, is to be married Jan. 17th (inst.), to Miss Wurzback, of that city.

---

**Dr. G. W. Christian,** one of the best known physicians in Texas—recently at Houston—has removed to Austin, Texas.

---

**Dr. Horace C. Hall,** a Texas graduate, until recently at Cancer Hospital, New York City, has returned to Texas and settled at Dallas.

---

**Dr. M. L. Berg,** long time a leading physician of Laredo, Texas, has removed to New York City, and is located at 309 W. 57th street.

---

**Patrons of the Journal**—subscribers and advertisers—are requested when remitting by local checks to add ten cents for collecting. Don't neglect it. Better send P. O. money order or express order.

---

**Married.**—At Mineral Wells, Texas, Nov. 28th (ult.), Dr. C. B. Williams, of Durant, I. T., to Miss Alice Raines, neice of Dr. and Mrs. C. B. Raines, of Mineral Wells. Dr. and Mrs. Williams will reside at Durant.

---

**Dr. Joseph Mullen,** of Houston, Texas, has just returned from the continent, where he attended the ninth International Congress of Ophthalmology, at Utrecht, Holland. He also spent three months at the clinics of Utrecht, Berlin, Vienna and Paris.

---

**Dr. J. D. Westervelt, Sr.,** died suddenly at his home in Corpus Christi, Dec. 16th, ult. He was a prominent and popnlar physician, and his death is a great loss to the profession of Texas. His son, Dr. J. D. Westervelt, Jr., is a prominent practitioner of Dallas.

---

**The New Orleans Polyclinic,** thirteenth annual session, opens November 30, 1899; closes May 10, 1900. Every inducement in clinical facilities for those attending. The specialties are fully taught. Further information, New Orleans Polyclinic, New Orleans, La.

**Doctor wants to sell** practice and residence for $675 cash, in black land prairie railroad town. Residence new; cost $800. Good thrifty American, German and Bohemian farmers. Collections 80 per cent. One other physician. Address "Doctor," Box No. 34, Buckholts, Texas.

---

**Dr. F. C. Ford,** of Houston, late of the Texas volunteer contingent in Cuba, has been appointed by the governor of Texas Medical Director of the First Division of the Texas State Volunteer Guard, with rank of Lieutenant-Colonel of staff corps. Colonel, the top of the morning to ye!

---

**The Journal American Medical Association.**—Dr. Simmons is making a most excellent journal of the organ of the Association. The "General Index," in the Dec. 30th number, alone is worth the subscription price. By the bye, membership in the Association ($5 a year) entitles one to the *Journal* free.

---

**Dr. D. L. Peeple,** of Navasota, late Surgeon of Second Texas regiment, Cuba contingent, U. S. V., has been appointed Surgeon to the First Brigade, State Volunteer Guard, with rank of Major. How are *you*, Major? And Dr. A. B. Kennedy has been appointed Surgeon Second Brigade, rank of Major. We tip our hat to the Major.

---

**A doctor wishes to sell** a small, new, well selected stock of drugs in a rapidly growing railroad town of eight or nine hundred inhabitants. Only drug store in the town. The doctor has no time to devote to drug business, on account of practice. A "gold mine" to the right man. Address R. E. B. Bledsoe, M. D., Somerville, Texas.

---

**One** of the JOURNAL's most valued advertising patrons, who has used our pages continuously for fourteen years, in renewing for 1900, writes: "We shall, of course, continue to advertise with you, and hope to do so for many years to come. We feel that it would be *utterly impossible to do any business in Texas* unless we were one of the red-back's adherents." That tells the tale. They are not doing it for fun.

---

**Dr. W. M. Wells,** late of Vicksburg, Miss., has settled in Corsicana, and engaged in the practice of medicine. The writer knows Dr. Wells well, and takes this occasion to congratulate the people and the medical profession of Corsicana on the acquisition of so estimable a gentleman and excellent physician as we know Dr. Wells to be. To the brethren we will say, he is worthy in every way of the respect and esteem of the guild, and the right hand of fellowship should be extended to him.

---

**Blood Dyscrasia.**—My attention having been called to a little pamphlet entitled "Blood Dyscrasia," I read it with much interest. Battle & Co., St. Louis, have put upon the market (and advertise only through the medical press and to physicians) a preparation of the active principles of *Echinacea* and *Thuja*, called "Echthol." It is a powerful antipurulent and fills a wide range of therapeutic indication, being especially useful in carbuncle, boils, ulcers, sores, skin diseases—in fact, in all conditions where "impoverished blood" exists, and a tendency to suppuration. We advise the red-back's readers to look into the claims of this remedy. Send a postal card to Battle & Co. and ask for a copy of the pamphlet and a sample of Echthol, and mention this notice.

---

**At N. O. Polyclinic.**—The following physicians were registered at the New Orleans Polyclinic in December: W. H. Alexander, Ivanhoe, Texas; H. H. Barnacastle, Haughton, La.; T. B. Bass, Terrell, Texas; W. H. Baucum, Millorton, La.; Chas. M. Blair, Rosenberg, Texas; C. M. Cocker, Mannsdale, Miss.; Ralph Hopkins, New Orleans, La.; J. S. Horsley, Jr., West Point, Ga.; D. A. Hutchinson, Nashville, Ark.; J. T. Jones, Jonesboro, Texas; O. S. Justice, Wetumpka, Ala.; E. A. Lee, Aspermont, Texas; L. Mantooth, Tupkin, Texas; W. H. O'Banion, Tilmon, Texas; W. H. Oliver, Bryan, Texas; R. E. Patrick, Tynwood, Miss.; Frank D. Piner, Denton, Texas; J. Pickard, New Orleans; H. O. Smith, Hamilton, Texas; R. A. Sterrett, Beckville, Texas; T. L. Treadaway, Lilac, Texas; S. C. Waller, Haynesville, La.; A. S. Warwick, Stanton, Va.; W. H. Williams, Dothan, Ala.; D. L. Wood, Burgess, Texas.

---

### Congress on "Tuberculosis and Its Modern Treatment."

It has been decided by the management of the Medico-Legal Society, to devote an extraordinary session to "Studies on Tuberculosis, Its Management and Modern Treatment," on the third Wednesday of February, 1900, at a regular meeting in this city, to open a full investigation and discussion of the whole subject, and to invite the leading American scientists and specialists to contribute papers, and unite in the discussion of this subject, and the most advanced and modern treatment of tuberculosis.

The following questions have been decided upon to be submitted for this discussion:

1st. Special hospitals and sanitariums, their construction and operation.

2d. What are the most successful methods of treatment?

3d. Individualization of certain forms of tuberculosis, its importance and necessity.

4th. Is change of climate a necessity for successful treatment?

5th. Should the use of anti-toxines in tuberculosis be condemned from a purely scientific point of view?

It is proposed to announce, in the program, the name of one or more experts, who will submit a paper upon each of these questions.

As a large number of the specialists are willing to take part at the opening meeting, after the dinner at the opening session, at 7 p. m., on the third Wednesday, it is proposed to continue the Congress the next day, so as to make the discussion full and complete.

It is proposed not to limit the titles of papers to those stated questions, in case an author desires to submit his views upon any other germane subject that he prefers.

The great interest in this subject, on both sides of the Atlantic, which also has been given great prominence in the Paris Congress of 1900, will make the movement of great interest, not only for the profession, but also the general public. Members and others who are willing to take part are requested to immediately write to either member of the committee, upon which of these questions they will submit their views prior to the first of February next, if possible, so that the same may be printed and submitted to the others who are to take part, in advance of the meeting, as is the custom in foreign countries.

The following committee has been named by the Medico-Legal Society, to act as a Committee of Arrangements for this Congress, with full power, and the profession is desired to co-operate by contributing papers and to taking part in the discussion, and to advise either member of the committee as early as possible.

Thomas Bassett Keyes, M. D.,
98 State St., Chicago, Ill.
J. Mount Bleyer, M. D.,
460 Madison Ave., N. Y.
Clark Bell, Esq.,
39 Broadway, N. Y.

—*Advance Sheets Medico-Legal Journal, N. Y.*

